# Protocol for process evaluation of integration of mental health into primary healthcare in two states in Nigeria: the mhSUN programme

**DOI:** 10.1192/bjo.2021.7

**Published:** 2021-02-15

**Authors:** Julian Eaton, Yusuf Akande, Uchechi Onukogu, Emeka Nwefoh, Taiwo Lateef Sheikh, Ekpe Essien Ekpe, Oye Gureje

**Affiliations:** Centre for Global Mental Health, London School of Hygiene and Tropical Medicine, UK; Research Unit, Department of Clinical Services, Federal Neuropsychiatric Hospital Kaduna, Nigeria; Department of Clinical Psychology, Federal Neuropsychiatric Hospital Calabar, Nigeria; Mental Health Department, CBM Country Office, Nigeria; Department of Psychiatry, Ahmadu Bello University College of Medical Sciences, Nigeria; Department of Clinical Services, Federal Neuropsychiatric Hospital Calabar, Nigeria; Department of Psychiatry, University of Ibadan College of Medicine, Nigeria

**Keywords:** Mental health services, primary care, low- and middle-income countries, process evaluation, Nigeria

## Abstract

**Background:**

Current international recommendations to address the large treatment gap for mental healthcare in low- and middle-income countries are to scale up integration of mental health into primary care. There are good outcome studies to support this, but less robust evidence for effectively carrying out integration and scale-up of such services, or for understanding how to address contextual issues that routinely arise.

**Aims:**

This protocol is for a process evaluation of a programme called Mental Health Scale Up Nigeria. The study aims are to determine the extent to which the intervention was carried out according to the plans developed (fidelity), to examine the effect of postulated moderating factors and local context, and the perception of the programme by primary care staff and implementers.

**Method:**

We use a theoretical framework for process evaluation based on the Medical Research Council's Guidelines on Process Evaluation. A Theory of Change workshop was carried out in programme development, to highlight relevant factors influencing the process, ensure good adaptation of global normative guidelines and gain buy-in from local stakeholders. We will use mixed methods to examine programme implementation and outcomes, and influence of moderating factors.

**Results:**

Data sources will include the routine health information system, facility records (for staff, medication and infrastructure), log books of intervention activities, supervision records, patient questionnaires and qualitative interviews.

**Conclusions:**

Evidence from this process evaluation will help guide implementers aiming to scale up mental health services in primary care in low- and middle-income countries.

Increasing access to evidence-based mental healthcare, or reducing the treatment gap, is a central priority of global mental health.^1^ In low- and middle-income countries (LMICs), reform of overly centralised services has focused on delivering mental health interventions in community or primary care settings,^2^ making use of existing infrastructure and systems where possible, for efficiency, sustainability, local ownership and equity.^3^ In the past 20 years, a substantial body of evidence has been established demonstrating the efficacy in terms of clinical, social and disability outcomes of programmes that use such models.^3,4^

A key concern of any global health field is the degree to which recommended approaches are acceptable, feasible or effective in different settings. The mandate of the World Health Organization (WHO) is, in part, to make available normative guidelines that can be applied in different settings, and the mental health Gap Action Programme (mhGAP) was developed for this purpose.^5^ Despite the fact that relevance to low- and middle-income settings was a clearly stated consideration of the mhGAP Intervention Guide,^6^ and there is a wide range of reports of mhGAP use in formal and grey literature, including a systematic review,^7^ there remains a question as to local applicability of the guidelines in the vastly different contexts in which they are to be applied.^8,9^

Although evidence on outcomes is essential to demonstrate the efficacy of mhGAP-based interventions, most evaluations in global mental health have been limited in terms of fidelity testing or formal analysis of the role of context, despite the complexity of such interventions, and uncertainty in relation to causality.^10^ Most studies have either been either formative in nature or carried out with tightly managed experimental methods, such as randomised controlled trials. Cost-effectiveness evaluation,^11^ and some assessment of client acceptability and feasibility,^12^ have generally been the main methods of additional exploration of these wider questions, usually as a part of larger outcome studies. More recently, there has been a dedicated process evaluation of integration of mental health into primary care in Mexico^13^ and India^14^ (part of the Programme for Improving Mental Health Care (PRIME) programme).

There remains a gap in terms of understanding mechanisms of impact, the role of local context and what factors contribute to effective integration of mental health services into primary care in low-income settings.^15,16^ In fact, the WHO mhGAP Operations Manual was not published until 2018,^17^ a full 8 years after the mhGAP Intervention Guidelines. Although the mhGAP Intervention Guidelines followed detailed methodologies for evidence use,^18^ this was not the case for the Operations Manual, reflecting the far less robust level of empirical knowledge about effective processes of implementing services, even after such a long period of field experience. This process evaluation aims to contribute to a stronger theoretical basis to guide translation of international evidence-based guidelines and consensus like mhGAP into effective implementation in the field.

## The Mental Health Scale Up Nigeria programme

The Mental Health Scale Up Nigeria (mhSUN) programme was developed in collaboration between the University of Ibadan, State Ministries of Health in Kaduna and Cross River States, Federal Ministry of Health (including two Federal Neuropsychiatric Hospitals in the states) and CBM, an international development agency.

The programme used the WHO mhGAP as a basis for integration of mental health into state primary care services, and was aligned to the National Mental Health Policy in Nigeria. In addition to the model of service provision, relevant factors deemed as necessary to maximise impact and sustainability were considered in programme design; for example, governance, population engagement and health system components such as human resource capacity, supervision and referral, health information systems and medication availability. It is described in detail in a previous paper,^19^ and its components are listed below (Table 1). See also Supplementary File 1, available at https://doi.org/10.1192/bjo.2021.7, for a summary of the core considerations in designing the intervention.

**Table 1:** Research questions, and linked data sources and analysis approach

Research question primary	Domains to be investigated	Data sources	Data analysis approach
1. To what extent does a programme of mental healthcare integrated into typical primary health services in low resource settings, adapted to the local context (the mhSUN intervention), adhere to its original design in implementation (fidelity)?	Fidelity and dose: Performance of core components of the mhSUN intervention See Table 2	See Table 2 Activity log books Facility checklist (staffing, medication available, etc.) Supervision forms	Comparison of standards documented in mhSUN Operations Manual, with actual implementation achieved at individual, facility and systems levels Descriptive analysis
	Coverage (see below): Contact Adequate Effective Equity	Health information system (service use data) Patient clinical records Existing prevalence data	Proportion of total sample meeting coverage criteria (see below)
	Perspectives of staff and patients, and providers of service model and implementation	Qualitative interviews with staff and patients	Inductive qualitative analysis to ascertain perceived moderators and contextual factors
Secondary research questions			
2. What is the effect of the key moderating factors hypothesised on the effective implementation of the model?	Putative moderating factors derived from theory (see Fig. 2), and from Theory of Change map^19^: Participant responsiveness/satisfaction Resources Recruitment/service uptake Model description and communication	See Table 3 Facility case study Cohort questionnaire Qualitative interviews Training logs (mhGAP) Activity logs (e.g. awareness raising)	Logistic regression Analysis to compare outcomes with and without moderating factor of interest Qualitative interviews to explore impact of moderating factors on outcomes
3. What are the contextual factors that promote or frustrate optimal service delivery, access and maximum coverage?	Contextual factors in local service and political environment	See Table 3 Situation analysis Facility case study	Qualitative interviews to understand perceived importance of contextual factors on implementation
4. How do patients use the service, and what is the level of accessibility and acceptability of the service for patients, carers and staff?	Accessibility Distance travelled Costs (direct and carer opportunity costs) Other barriers to access Acceptability Cultural understanding and adaptation Awareness-raising effectiveness Perception of patients and staff	Cohort questionnaire: PACIC Qualitative focus group Discussions with patients, carers and staff	Distance and cost form part of assessment of moderating factors (below) Comparison of PACIC scores across sites to assess service use satisfaction against intended programme attributes Qualitative interviews to assess both extent of accessibility and acceptability, and granular perceptions of the service

mhSUN, Mental Health Scale Up Nigeria; mhGAP, Mental Health Gap Action Programme; PACIC, Patient Assessment of Chronic Illness Care.

**Fig. 1:**
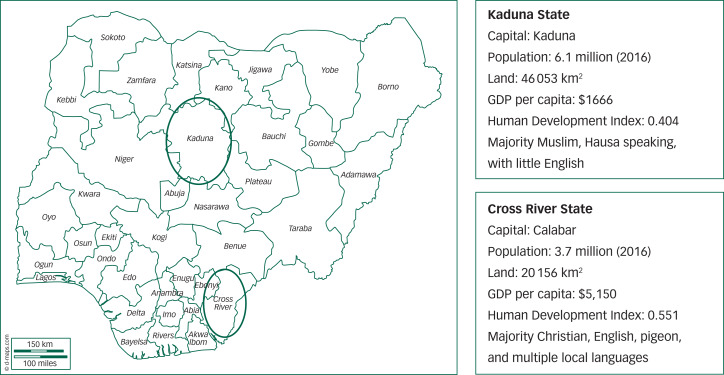
Map of Nigeria showing the research sites Kaduna State and Cross River State. The Gross Domestic Product (GDP) is a monetary value of all goods and services produced in a given period. The Human Development Index (HDI) is a measure combining dimensions of health, education and standard of living.

Two sites for the research were chosen to capture the major differences between the north and south of the country (Fig. 1). Cross River State is in the southern Niger Delta, with a predominantly Christian population made up of several small linguistic and ethnic groups. Kaduna, in the north of the country, instead has an influential religious structure among the strongly Muslim Hausa-speaking population. Kaduna State has a lower Human Development Index (0.404 compared with 0.551 for Cross River State), reflecting lower average income, poorer access to healthcare and lower education levels. With very different cultures and traditions, in Nigeria, it is considered essential to demonstrate an understanding of both Northern and Southern contexts, to show potential broad applicability of any proposed system reform (and to meet the political imperative of so-called ‘national character’). Both sites have a Federal Neuropsychiatric Hospital, with a leadership committed to applying the national policy with respect to establishing access to mental healthcare at primary health level, and a basic health system structure that reflected global conventions. However, in both cases, decades of under-resourcing have left a weak primary care system, influenced by vertical programmes funded by international donors, taking priority-setting, at least in part, out of the hands of local authorities.^20^ This has tended to undermine mental and other non-priority areas of health, including capacity to deliver at primary care level, whatever policy dictates.^21^

## Method

The aim of the process evaluation is to build a practical evidence base to support the appropriate scale-up of acceptable, effective and accessible mental health services in LMICs, based on learning from the Nigeria context.

### Theoretical basis for the process evaluation

**Fig. 2:**
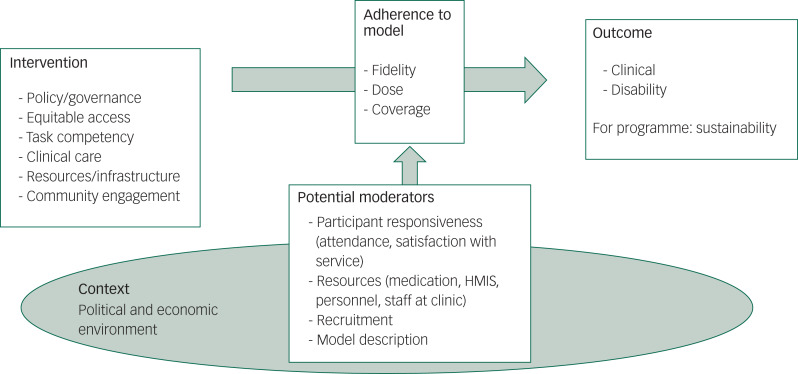
Conceptual framework for evaluation of the Mental Health Scale Up Nigeria (mhSUN) intervention. HMIS, health management information system.

We have drawn on a rich literature providing theoretical frameworks for evaluating implementation of health reforms; for example, Linnan and Steckler's work,^22^ based on large public health trials in the 1990s,^23,24^ further developed by Carrol et al^25^ and Hasson^26^ (see Fig. 2 below). Much of this work is synthesised in the Medical Research Council's ‘Guidelines for Process Evaluation of Complex Interventions’.^27^ Given the poor record of sustainability of externally funded programmes in international development, we also draw upon two other influential frameworks, Reach, Effectiveness, Adoption, Implementation, and Maintenance^28^ and Normalisation Process Theory,^29,30^ as they highlight essential relevant factors to be considered in recognising context, and sustainability and acceptability issues.

Literature around strengthening primary healthcare, often now linked to achieving universal health coverage, has a long tradition of measuring outcomes and some process factors, including in LMICs.^31^ The WHO and global funders have developed national and global scorecards,^32,33^ typically using the structure-process-outcome model as a framework.^34^ Well-established work in HIV services probably has the most relevance for mental health integration because of parallels in being a stigmatised chronic disease, often requiring long-term care and support. Less work has been done in the non-communicable disease sector, and in the contexts in which we are working, to date, most research has been associated with specific global health priorities such as vaccination,^35^ child and maternal health,^36^ and bed net distribution for malaria control.^37^ Important work has been carried out to explore the integration of mental health into primary care in LMICs, such as the PRIME^38^ and Emerald projects,^39^ and this intervention and study makes use of many of the approaches developed in this work.

The basis of the intervention itself was developed using Theory of Change^40^ (ToC), and was documented in a mhSUN Operations Manual (see Supplementary File 2). ToC uses a participatory approach to map the steps by which we hypothesise achieving a desired outcome, documenting the associated assumptions and contextual issues that are relevant for each step. ToC was used in a similar way in the PRIME programme, which highlighted additional advantage of this approach; alongside drawing on their expertise, the participation of key stakeholders in the ToC workshop recognise and value their contribution to the change process, gaining buy-in and commitment.^41^ This may help achieve project outcomes by gaining future political support and investment from health system leaders, motivation for key staff, or trust from patients and families.

### Methods, data sources and analysis

These theoretical frameworks and the ToC map for this intervention provides a basis for identification of key elements of the process of change researchers may wish to investigate further, by asking research questions relevant to the assumptions at key steps. Establishing and documenting such putative mechanisms for change and the effect of contextual factors in advance allows for subsequent hypothesis testing during the process evaluation.

We will use a mixed-methods approach with data from individual, facility and system levels and a variety of sources (below). Analysis and interpretation will include triangulation between quantitative and qualitative data, measuring the degree to which the intervention was implemented as intended, the influence of context and moderating factors, and providing an opportunity to gain insights from participants in the implementation.

Data sources include:

- A situation analysis carried out at the programme development stage.
- A facility case study comprising quarterly evaluation of facilities, including a facility checklist of human resource presence, medication availability and infrastructure, as well as record of changing context.
- Log books of intervention elements such as governance processes, advocacy meetings, awareness campaign, training and supervision.
- Routine health information system recording service use statistics, disaggregated by primary diagnosis and gender, and patient clinical records.
- Supervision record forms documenting monthly supervision visits, incorporating assessment of quality of care provided.
- Cohort of patients comprising a sample of patients with each of the three major diagnoses of epilepsy, psychosis and depression (the priority conditions for the service) will be interviewed on entry to the service and at 6 months. Data includes validated questionnaires on clinical, human rights and disability outcomes, patient satisfaction and service use (including costs). A conservative sample size of 200 participants per diagnosis has been calculated to achieve sufficient power to measure change in outcomes on clinical and disability measures. This was based on outcomes of similar studies exploring integration of treatment of depression, psychosis and epilepsy in primary care in LMIC settings.^42,43^
- Qualitative data collected through in-depth key informant interviews, and observation notes kept during site visits. Processes to engage and gain buy-in from relevant actors are built into the governance of mhSUN, so we expect to be able to obtain consent from key actors.

Data collection is carried out by research assistants and managed by a research coordinator in each site. The research team is attached to the Federal Neuropsychiatric Hospital where the mhSUN programme is managed, but functions independently of the implementing teams in terms of roles. Data collection forms were developed and tested for the facility data, supervision records and log books for activities. Mental health elements also had to be incorporated into the routine Primary Health Care data collection forms. as this was not previously included. The cohort questionnaire is programmed into a mobile application (Mobenzi Android v4.15.0; www.mobenzi.com), allowing efficient and secure data collection, storage and transfer. Such applications have the advantage of flexible use of skips and assured completion of questions, making data collection efficient, reducing missing data and increasing data quality.

We now outline background, methods and analysis for each research question in greater detail.

### Primary research question 1: intervention, adherence (fidelity, dose) and coverage

The intervention has been described, after development with ToC, including its essential components and the theoretical underpinning of these components.^19^ The major components of the model, as described in the Operations Manual, are summarised in Table 2. This is used as a basis for training and is available to implementers, and it is against this that fidelity will be measured.

**Table 2:** Components of the mhSUN intervention, and data sources for assessment of fidelity

Component (documented in mhSUN Operations Manual)	Expected interventions	Source of data for assessment
1. The policy and legislative framework for service reform	Alignment to policy in project development Engagement with political/health leaders	Operations Manual Activity log books
2. The structure and management of the service, governance	Establishment of Steering Committee and 6 monthly meetings Monthly programme meetings	Activity log books Meeting participant lists
3. Equity and access to the service	Community awareness programme	Health information system (disaggregated data)
4. Human resources and capacity building	Identification of personnel Core mhGAP training 6-monthly refresher training	Training records Facility checklist
5. Supervision and maintaining quality of care	Supervisor training Monthly supervision from federal hospital each month	Supervision forms
6. Clinical contact and referrals	Identification of clinic space Availability of trained personnel Links to referral hospitals	Facility checklist
7. Maintaining contact, strategies to deal with non-attenders	Community champions	Patient clinical records Health information system
8. Availability of medication	Advocacy and engagement with state on drug supply chain	Facility checklist Facility case study Drug Revolving Fund records (Calabar)
9. Raising awareness, and increasing uptake of the service	Community awareness programme Community champions	Activity log books Health information system
10. Record-keeping and health information system	Development and use of mental health information system	Health information system
11. Social integration and rehabilitation	Link to community champion Community awareness programme	Patient clinical records

mhSUN, Mental Health Scale Up Nigeria.

Following descriptive analysis of these data, comparing expected with actual intervention components, we will collect qualitative data as a means of documenting the perspectives of the key actors involved (healthcare providers, implementers and leaders in the community and health system), to help interpretation of quantitative results.

The interview topic guide for the qualitative data will be situated within the theoretical models employed, and will be based on examination of the assumptions (leading to points of enquiry for research) made in the ToC. Inductive thematic analysis of interviews will be carried out following transcription, and the results used to triangulate with quantitative results on hypothesised moderators and contextual factors.

The data generated can also be used for subsequent deductive analysis, such as using structured frameworks like the Consolidated Framework for Implementation Research,^44^ allowing for greater comparability across contexts, and application to structured and practical processes of translation of research to practice. Observational data collected throughout the implementation (field notes and notes made by implementers in the log books) will also be used to inform the interpretation of the quantitative data.

It should be noted that dynamic adaptation to context and changing circumstances is essential in successful implementation of any intervention, especially when in a novel environment.^45^ This will be recorded throughout the intervention, particularly in the observational notes and qualitative interviews.

### Coverage/reach

This refers to the proportion of the target group are affected by the intervention (see Supplementary File 3 for a process flow diagram). This is a key consideration for efficiency and equity, and is of great interest to decision makers in government – the target of our research results. Three levels of coverage will be measured.^46^ The first level is contact coverage. Routinely collected data available at the clinic will be used to measure clinic attendance for mental disorder. Published mental disorder prevalence data, weighted for the demographics of the local community, will then be used to obtain the denominator for calculation.

The second level is adequate coverage. To assess the proportion of the people receiving an adequate intervention, the cohort questionnaire included a measure of patient satisfaction with service (Patient Assessment of Chronic Illness Care (PACIC) questionnaire^47^). We will compare the PACIC results with the supervision report, which scores patient treatment on a scale based on whether assessment, diagnosis, treatment and appropriate referral were carried out by attending clinicians (general Primary Health Care nurses). Although specific tools to assess quality of clinical care in Primary Health Care in LMICs have been developed (e.g. the ENhancing Assessment of Common Therapeutic factors tool^48^), this is prone to the Hawthorne effect. We have tried to avoid this by assessing anonymous patient notes for quality of clinical care.

The third level is effective coverage. This is defined as the proportion of patients achieving recovery based on 6-month clinical (symptom reduction) and disability outcome data (WHO Disability Assessment Schedule 2.0, which has been used extensively in similar studies of mental healthcare reform,^49,50^ including in Nigeria^51^). For depression, we used the Patient Health Questionnaire-9, which has been validated and used widely in Nigeria;^52^ for epilepsy, we used a count of seizures in the previous month. Clinical Global Impression–Severity score was used for all participants entering the cohort study, and this was used as a proxy for psychosis, where a good correlation has been reported for more complex change measures.^53^ In addition, quality of training is assessed with standard mhGAP pre-and post-questionnaires, and competency forms part of supervision reports.

In addition, issues of equity (a component of reach/coverage) will be assessed by routinely collected service use data, disaggregated by age, gender, socioeconomic status, diagnosis and distance from the service.

### Research questions 2 and 3: context and potential moderators

#### Context

A detailed situation analysis at state and local government levels was carried out in each state during design of the intervention. This will be reviewed and summarised with respect to potential contextual factors that could affect the implementation. In addition, each quarter, as part of the facility case study, personnel document any environmental factors that they feel may have influenced implementation or outcomes of the programme. Both the situation analysis and facility case study borrow extensively from the work carried out in the PRIME programme,^54^ which shared many contextual characteristics with mhSUN. The importance of these contextual factors will be further explored by key informant interviews, recognising that this is a dynamic process, with the programme being affected by, and affecting, the local environment.

#### Moderators

Postulated moderators for effective implementation were derived from theoretical frameworks and the ToC developed for this project. This process drew upon local experience to highlight what factors might influence outcomes. These were documented as preconditions for achieving the different steps of change in the mhSUN ToC (and associated indicators assigned to them) (see previous mhSUN publication^19^):

- Participant responsiveness/satisfaction: Does quality of treatment or staff clinician behaviour influence service use patterns?
- Resources: What was the effect of availability/lack of availability of human resources, health information systems, medication, clinic infrastructure and other resources to implementation of the programme as per time/output expectations?
- Recruitment/service uptake: What factors increased service use and the number of appointments patients attended? What was the effect of recognised barriers to access (costs, distance) and of the awareness programme?
- Model description and communication: Did staff feel they understood the model and feel involved in its development and adaptation? How do base training and refresher sessions improve staff knowledge and improve treatment?

Information on putative moderating factors influencing implementation have also been included in data to be collected (Table 3).

**Table 3:** Potential moderating factors and data sources

Potential moderating factors	Data source^a^
Participant responsiveness/satisfaction	
Experience of service	Cohort questionnaire (PACIC)
Participant experience	Qualitative interviews with patients and staff
Quality of clinical care	Supervision reports
Resources	
Availability of medication	Facility case study Q6
Availability of key staff at clinic	Facility case study Q4
Integration of mental health into health information system	Facility case study Q1c
Condition and availability of private clinic room	Facility case study
Recruitment/service uptake	
Cost of service	Cohort questionnaire (CSRI)
Socioeconomic status	Cohort questionnaire (HES Nigeria)
Distance from facility	Cohort questionnaire (patient records)
Timing of awareness programme	Awareness programme log book/service use data/HMIS
Model description	
Training provision	Training logs (basic and refresher training)
Training outcomes	Pre- and post-training questionnaires
Service provider understanding of model	Qualitative interviews with service providers

a. See supplementary material for facility case study and cohort questionnaire details.

PACIC, Patient Assessment of Chronic Illness Care; CSRI, Client Service Receipt Inventory; HES Nigeria, Household Expenditure Survey; HMIS, (routine) health management information system at Primary Health Care.

We will use descriptive analysis of facility-level data and logs (training attendance, medication availability, staff presence, etc.), using data at different time periods to compare outcome with and without the relevant moderating factors of impact of awareness-raising programme on service uptake; availability (or not) of medication, and any correlation with service provision and uptake; and actual rate of programme components bedding down (compared with initial plans and expectations).

In addition, as the programme is being carried out in two states (and within seven and eight districts within each of these), we will be able to examine the effect of differences in implementation across location; for example, the impact of localised policies on drug distribution on availability, the effects of support from local political and health leadership, and the influence of insecurity on implementation in different districts.

For data collected through the cohort questionnaire, we will be able to summarise data on costs, service use and other cross-sectional information, and carry out logistic regression analyses (using Stata for Windows version 9 to generate odds ratios) to identify associations between different factors and outcomes, such as looking at whether a group reporting easy versus barriers to access to a clinic had better outcomes.^55^ Although such analysis risks being underpowered, we will explore whether the results confirm or refute our hypotheses, and we will be triangulating this with the qualitative work, allowing us to explore potential mechanisms, using the experience and expertise of key informants.

### Research question 4: accessibility and acceptability

Ensuring accessibility and acceptability was a consideration throughout the design of the intervention, as demonstrated by the use of ToC and extensive engagement with local authorities. Language and cultural understanding are a consideration throughout in the research and programme elements, as shown by awareness-raising and local concepts of mental illness being incorporated into communication materials. Accessibility and acceptability will be assessed through the cohort questionnaire (PACIC), and in qualitative interviews with patients, carers and healthcare staff, drawing on similar research.^56^ Focus group discussions will be used both in qualitative evaluation of acceptability of the intervention, and in triangulating with factors deemed to influence outcomes, as acceptability is a potential moderator (part of Participant Responsiveness (see Fig. 1).

### Strengths and limitations of the study design

The study is a pre-planned process evaluation exploring local contextual factors, filling a gap in literature for mental health integration into primary care that is mainly of outcome evaluations to date. There is a strong theoretical basis for the intervention, based on international guidelines (WHO mhGAP) that are widely accepted normative standards. Local experience and context are considered with a ToC approach. The use of mixed methods, with data capture through routinely collected key data and through purposive quantitative and qualitative methods, allows triangulation of results from different sources, testing assumptions from the ToC and allowing reflection by key actors of potential mechanisms. Weaknesses in routine data collection will be managed with provision of additional statistics forms for mental health and related processes.

A key limitation is the many external factors that are likely to influence the implementation of the mental health intervention in a generally weak and fragile system, and a context of political uncertainty with a history of communal violence. However, the research aims to inform practice in just these settings and a real-world evaluation is of more value than a highly controlled experimental design. These factors will be captured and their impact interpreted through the study design used.

### Ethics and dissemination

Ethical approval has been given by the ethical review boards of London School of Hygiene and Tropical Medicine (Ref: 11056 /RR/5812), Ibadan University and the two Federal Neuropsychiatric Hospitals in Calabar and Kaduna. Consent will be obtained from all participants interviewed in the quantitative or qualitative components of the research. Extensive engagement was carried out with the local primary care services, including gaining permission from the State Ministries of Health (Commissioner, Primary Care Director), during programme design. At the local government area level, programme staff visited the local government Chairman and discussed the research with the Supervisory Councillor for Health, and each Primary Health Care unit head and relevant local government area monitoring and evaluation leads. A Memorandum of Understanding was signed with each state Ministry of Health and local government area, which gave formal authority for the work. In addition to these local health officials, local traditional leaders, tradition healers and religious leaders were regularly visited throughout the period of the study.

In Kaduna, most data will be collected in Hausa, and surveys questionnaires have been translated and back-translated accordingly. In both sites, research assistants speak the range of local languages. Interviews will be transcribed and de-identified. The number of key informants will be relatively low, but we still do not expect it to be possible to identify individuals. Recognising language and literacy issues, clear participant information sheets have been developed, and research assistants have been taught to share relevant information clearly (verbally) and to respond to queries. Data will be securely stored throughout the process, including through the use of mobile digital data collection, and is only accessible to the research team members who possess a password (primary investigators and research coordinators at sites). All data for the cohort is anonymised at the point of collection. Other data collected at the sites will be stored by the research coordinator in a locked office, and on a password-protected computer. It will be sent at regular intervals to a server established for this purpose at London School of Hygiene and Tropical Medicine.

Safety of research and implementation teams is a major consideration in this project because there is a history of communal violence in both states. Local districts were chosen to avoid this as much as possible, but it is felt important to carry out research in these settings that represent a large proportion of Africa, where mental healthcare is most needed and least available. Procedures will be put in place to minimise risk, guided by site teams and based on local knowledge of the risks at any particular time. This process (used during the research phase) will reflect real decision-making processes used in implementing programmes in practice.

As with other mental health service reform programmes at a pilot scale, the study's aim of influencing service planners is the main justification for a process evaluation study. The study aims to directly inform the investment and policy decisions of political and health leaders, and the Federal Ministry and State Ministries of Health in Nigeria are the primary target for dissemination, but results would be of interest to other governmental and civil society implementers, particularly in wider sub-Saharan Africa and in LMICs more broadly.

Resources have been allocated to share results with the National Mental Health Action Committee of the Federal Ministry of Health and in meetings at state Ministries of Health, as well as directly with those involved in the study in community meetings.

We plan to publish the results of this work in two separate papers (in addition to the development paper already published): research questions 1–3, where analysis will draw together fidelity and outcomes with moderators and contextual factors; and a separate paper on accessibility and acceptability of the intervention.

In addition to peer-reviewed academic literature, we will use networks of global mental health, such as the Mental Health Innovation Network (www.mhinnovation.net), which is widely accessed by implementers and policy makers. We expect to be able to provide the mhSUN programme implementers with learning that they may apply to refinement of the model to facilitate improved outcomes and sustainability, and will hold feedback sessions to local implementers and relevant stakeholders. In this way, we hope that the results of the work will be of value both locally and more broadly.

## Data Availability

As a protocol paper, data availability is not applicable to this article because no new data were created or analysed in this study. However, any information related to the study protocol are available on request from the corresponding author, J.E.

## Supplementary material

For supplementary material accompanying this paper visit http://dx.doi.org/10.1192/bjo.2021.7. click here to view supplementary material
