# Supplementary material for: Protocol for process evaluation of integration of mental health into primary healthcare in two states in Nigeria: the mhSUN programme
Source: BJPsych Open. 2021 Feb 15;7(2):e54. doi: 10.1192/bjo.2021.7 (PMC8058900; doi:10.1192/bjo.2021.7)
Supplement: Supplementary file 1 [file bjosup.zip › S2056472421000077sup001.zip!/Appendix_1._Main_components_for_systems_reform_in_mental_health.docx]

**Appendix 1: Main components for systems reform in mental health**

The WHO Mental Health Gap Action Programme (mhGAP) started initially by publishing an Intervention Guide, recommending specific clinical interventions (almost all at the one-to-one clinical/patient level) for delivery in Primary Health Care. The presumption was that the system infrastructure that would support such an encounter, and enable the treatment offered and any follow-up to be delivered, would be in place. Initially, the nature of the reforms necessary were not clearly defined, but based on broad principles, borrowed from other fields such as HIV and the primary health care literature. WHO more recently published an mhGAP Implementation Manual, bringing together much of this information, much of it drawn from over 10 years of field-based practice and research trials since the original mhGAP Intervention Guide was published.

Here we present a summary of these concepts, which also underpin the mhSUN intervention.

| Deinstitutionalisation and shift to community-based mental health care | The realisation that almost all aspects of long-stay mental institutions are anti-therapeutic and often abusive, led to an international consensus that they should be closed in favour of community-based alternatives.^[1]^ Community-based generally refers to the idea of people living in their own homes, accessing health and social care from general services as needed, though some people with severe conditions may live in small group homes, but still with the aim of replicating the lifestyle and freedoms of the general population. While in many high income countries, this shift occurred in the 1980s and 90s, large psychiatric hospitals remain the mainstay of mental health services in many low and middle-income countries. |
| --- | --- |
| Balanced Care Model and the pyramid of services | Specialist hospitals do have a role in providing treatment for people who require temporary in-patient care during acute episodes of mental illness. The Balance Care Model^[2]^ recognises the need for the appropriate level and type of care being available for people when and where they need it. Services should be available at primary, secondary and tertiary levels of the system, with good referral between levels. Specific services are also necessary for children and adolescents, and for older people, in addition to specific services for different conditions, such as alcohol and drug services, perinatal care for women, and early intervention in psychosis. The WHO pyramid of services depicts this, highlighting that far more people will need care at the more basic (and less expensive) base of the pyramid. |
| Systems approach; | There are many components of the health system that play an essential role in successful service delivery and must be considered in reform. If one key element is not functioning well, there is likely to be a reduction in overall systems performance. WHO’s health system building blocks use this approach, and are made up of;  Leadership and governance  Service delivery  Health system financing  Health workforce  Medical products, vaccines and technologies  Health information systems |
| Integration into primary (and secondary care); | While historically mental health services have often been delivered separately from physical health care, there are no good reasons for this, and in fact this artificial separation is not only inefficient, but stigmatising, and tends to lead to poorer quality and less accessible mental health care.^[3]^ Instead, mental health services should be integrated into general health services at primary and secondary care. This allows use of general health system infrastructure, and general clinicians to deliver most basic care (as in other areas of health care). The strength of this approach can also be a challenge in resource poor settings when the quality and resourcing of these general services are weak. |
| Task shifting; | Based on the recognition that there will never be sufficient senior mental health personnel, Task Shifting (or Task Sharing) has been proposed as the major solution to human resource gaps. It is the main model employed in most service reform research projects carried out to date. |
| Collaborative, stepped care; | If relatively inexperienced staff with only a basic training are to provide good quality care, aligned to the best evidence and consensus best practice, they require ongoing expert support. Such provision of supervision and access to advice is called collaborative care. A complementary approach is to ensure that if a front-line worker feels that a particular case is too complex or not responding to the level of care within their competency, they should be able to refer the patient to a more experienced clinician. This is called stepped care, and is usually based on clearly defined competencies, and points for referral. mhGAP uses this approach. |

1. World Health Organisation and Calouste Gulbenkian Foundation. Innovation in deinstitutionalization: a WHO expert survey. World Health Organisation, 2014 Available from: <https://www.who.int/mental_health/publications/gulbenkian_innovation_in_deinstitutionalization/en/>
2. Thornicroft G, Tansella M. The balanced care model: the case for both hospital- and community-based mental healthcare. Br J Psychiatry. 2013 Apr;202(4):246-8. doi: 10.1192/bjp.bp.112.111377
3. WHO and Wonca. Integrating mental health into primary care. A global perspective. Geneva, World Health Organization and World Organization of Family Doctors, 2008
