# Supplementary material for: Protocol for process evaluation of integration of mental health into primary healthcare in two states in Nigeria: the mhSUN programme
Source: BJPsych Open. 2021 Feb 15;7(2):e54. doi: 10.1192/bjo.2021.7 (PMC8058900; doi:10.1192/bjo.2021.7)
Supplement: Supplementary file 1 [file bjosup.zip › S2056472421000077sup001.zip!/Appendix_2._mhSUN_Operations_Manual.doc]

**Mental Health Scale Up Nigeria**

**mhSUN**

**MODEL OPERATIONS MANUAL**

**and**

**RESEARCH STANDARD OPERATING PROCEDURES**

Table of Contents

Chapter 1: Background	3

Introduction to mhSUN	3

Chapter 2: Scientific and Technical Administration	5

Partners	5

Organizational structure	5

Research Group	7

Field implementation group	7

Personnel roles and responsibilities	8

Administration and Research	8

Chapter 3: The mhSUN Intervention	11

Introduction	11

1. The policy and legislative framework for service reform	14

2. The structure and management of the service	15

3. Equity and ensuring access to the service	18

4 Human Resources and Capacity Building	18

5 Supervision and maintaining quality of care	20

6 Raising awareness, and increasing uptake of the service	21

7. Clinical care and referrals	22

8 Maintaining contact, and strategies to deal with non-attenders	23

9. Availability of medication	23

10. Record-keeping and Health Management Information System	25

11. Social integration and rehabilitation	25

Chapter 4: Research Standard Operating Procedures	27

4.1 Facility Case Study	27

4.2 Routine Health Information from clinics	28

4.3 Activity Logs	28

4.4 Cohort Study	29

4.5 Qualitative Study	32

Chapter 5: Data Management Plan	34

Appendix I: Key to Theory of Change Map	39

Appendix II: Awareness-raising plan template	40

Appendix III: Draft cohort participant information sheet	42

Appendix IV: Draft cohort consent form	45

# Chapter 1: Background

## Introduction to mhSUN

The urgent need to provide accessible services to close the treatment gap for those with mental disorders in Africa is well recognized.^[1],[2]^ As part of the process of scaling up coverage of effective services to achieve this, packages of care that have been shown to work effectively in this context are necessary.^[3]^

This research will evaluate the Mental Health Service Scale Up in Nigeria programme (mhSUN). This intervention aims to increase coverage of accessible, affordable and quality mental health services in Nigeria by integrating mental health at primary and secondary levels. The WHO’s mhGAP programme is used as the evidence base for the clinical interventions used, which is in line with Nigerian mental health policy and strategy.

The programme is being implemented in one state in the South of the country (Cross River State), and one in the North (Kaduna State). In this way, some of the variety that is found in the country can be accommodated so as to understand how a programme can be made to work across the diversity of the country.

*Figure 1: Map of Nigeria, showing the two states of the mhSUN intervention*

In addition to the implementation of a pilot programme in 2 States and its evaluation, other specific interventions in the programme include:

- Training of mental health leaders in public health and advocacy skills through the mental health leadership and advocacy programme (mhLAP) in Ibadan, Nigeria
- Building capacity of researchers in the country through training, and interaction with international researchers
- Engaging with Federal and State Governments to advocate for greater public investment in mental health. This occurs at state level, for example through the State Steering Committee, and at Federal level through the National Mental Health Action Committee.

# Chapter 2: Scientific and Technical Administration

## Partners

**Department of Psychiatry, University of Ibadan** will co-ordinate the mhSUN project, build capacity of the leaders and train the trainers for mhGAP and will also participate in research M&E and model development. They will also liaise with international academic partners such as the London School of Hygiene and Tropical Medicine, who will provide technical support in research and M&E.

The Federal Neuropsychiatric Hospitals Kaduna and Calabar are the implementing institutions. They will lead the model development and integration into State and LGA structures and will be responsible for M&E data collection.

The Federal Neuropsychiatric Hospital, Aro will be advise on model development and M&E.

The Federal Ministry of Health (FMoH) will be the target for advocacy for scaling up of services and host the 6 monthly National Mental Health Action Committee meetings.

CBM mental health advisors will provide technical support in model development, research, M&E, advocacy and inclusion.

The CBM Global Advisor will be the lead on the research aspects of the project.

The CBM Nigeria Country Co-ordination Office will support University of Ibadan in co-ordination of the programme. It will be responsible for capacity building, receipt and disbursement of funds for activities to UI, and receipt of report from the partners, verification of same and transmitting to CBM Australia.

## Organizational structure

The programme is coordinated by University of Ibadan, and implemented by Federal Neuro-Psychiatric Hospitals in Kaduna and Calabar. CBM is the international partner providing technical and financial support (through funds provided by the Australian Government). There has been long-standing collaborations between these partners, mainly focusing on system strengthening, and support for self-advocacy by service users in Nigeria ^[4],[5]^.

The working organizational structure including all partners, and relevant lines of funds transfer and reporting are as follows:

## Research Group

The research evaluation of the mhSUN Programme is overseen by Julian Eaton as the main focus of his PhD at London School of Hygiene and Tropical Medicine.

*Advisory Group Members*

Supervisor: Dr Alex Cohen, LSHTM

Associate Supervisor: Professor Oye Gureje, University of Ibadan

Associate Supervisor: Dr Sujit Rathod, LSHTM

Member: Dr Daniel Chisholm, WHO

## Field implementation group

*University of Ibadan*

Director of mhSUN programme: Prof Oye Gureje

Project Coordinator: Woye Fadahunsi

Technical Advisor: Lola Kola

*Federal Neuropsychiatric Hospital Calabar*

CMD: Dr Okegbe

Lead Consultant: Dr Ekpe

Research Coordinator: Uche Onukogu

Research Assistants (5)

*Federal Neuropsychiatric Hospital Kaduna*

CMD: Dr T.L. Sheikh

Lead Consultant: Dr M. Abdulaziz

Research Coordinator: Yusuf Akande

Research Assistants (5)

*State Steering Committees*

On addition, each State site has a State Mental Health Steering Committee, made up of all relevant stakeholders in the State. This acts as an advisory and oversight Board for the project.

*CBM Country Office, Abuja*

Administrative support

Technical support from Dr Emeka Nwefoh, CBM Mental Health Advisor for Nigeria

**Meetings**

1. The State Advisory Boards meet every 6 months. Meeting agendas are prepared by the State teams, and minutes taken.
2. The research team meets for a telephone meeting every month (Coordinated by the University of Ibadan Coordination team.
3. The PhD Advisory Group are consulted by JE when relevant, and will meet annually (by telephone) as a minimum. JE will meet individual members (in Nigeria and London) regularly in person.

## Personnel roles and responsibilities in Administration and Research

**Programme Director (Ibadan)**

Duties and responsibilities

1. Provide overall leadership and guidance for the mhSUN programme implementation and associated research elements
2. Supervise team in Ibadan, and provide guidance for teams in Kaduna and Calabar

**Project Coordinator (Ibadan)**

Duties and Responsibilities

1. Manage the overall implementation of the mhSUN programme, liaising with the different partners. This includes support for the research elements of the programme
2. Organise and facilitate monthly team meetings by telephone
3. Other duties deemed important for effective and efficient implementation of the study as assigned by the Project Director

**Technical Advisors (Ibadan and CBM)**

Duties and responsibilities

1. Support the mhSUN programme by working alongside the medical and research teams to ensure that best practice is observed, and quality of the service is maintained.
2. To contribute to the design of the mhSUN research programme
3. Visit the field sites regularly (minimum 2 times a year), particularly during particular events such as training, reviews etc
4. Other duties deemed important for effective and efficient implementation of the study as assigned by the Project Director

**Consultant Psychiatrist lead (FNPH Calabar and Kaduna)**

Duties and responsibilities

1. To lead the mhSUN implementation as well as oversee research work in each Federal Neuropsychiatric Hospital.
2. To train, monitor and supervise the Research Coordinator for effective and efficient implementation and research data collection.
3. To contribute to the design of the mhSUN research programme
4. Participate in monthly mhSUN research telephone meetings and follow up on decisions taken at the meeting
5. Participate in analysis and writing up of research findings for publication

**Research Coordinator (FNPH Calabar and Kaduna)**

Duties and Responsibilities

1. To train, monitor and supervise Research Assistants for effective and efficient implementation and data collection activities of the mhSUN research study
2. Participate in collection of data for Facility Case Study, Cohort Study and Activity Logs for evaluation of mhSUN programme
3. To contribute to the design of the mhSUN research programme
4. Participate in monthly mhSUN research telephone meetings and follow up on decisions taken at the meeting
5. To ensure that all relevant materials (training materials, data collection, awareness raising) are ready and available for the proper implementation of the programme.
6. To provide project support as is necessary.

**Research Assistants (FNPH Calabar and Kaduna)**

Duties and Responsibilities

1. To contribute to the design of the mhSUN research programme
2. To follow the Standard Operating Procedures for collection of data and storing appropriately.
3. Visit the field to complete Facility Case Study, Cohort Study (recruitment, questionnaire administration, follow-up and finding patients who do not come for follow-up).

**Administrator (FNPH Calabar and Kaduna)**

Duties and Responsibilities

1. Collect relevant financial information to complete financial reporting expectations, including receipts, vehicle logs, inventories of equipment etc
2. Ensure proper procurement procedures are pursued for purchases, printing etc
3. Work with implementing team to ensure that funds are spent on time and according to budget
4. Report any issues to Lead Consultant so that they can be resolved quickly.

# Chapter 3: The mhSUN Intervention

## Introduction

This document outlines a model of service delivery aimed at demonstrating how coverage of quality mental health care services can be significantly increased in Nigeria. The model follows the basic principles of providing affordable, accessible and acceptable mental health care. This will be achieved within secondary (general hospital) and Primary Health Care settings, by integrating mental health into government services. This is supported by Federal Ministry of Health, State Ministries of Health, and specialist hospitals, as well as an international NGO, CBM International in a public-private partnership.

The model is based on;

- Review of current evidence and accepted best practice in scaling up mental health services in low income settings^[6]^.
- Adaptation of service models developed and tested in other parts of Nigeria
- Local situation analysis
- Participatory development process involving a variety of local stakeholders, including providers and users of services

During the process of development of the programme, a Theory of Change map was developed, which systematically looks at how the aim of the programme can be achieved by working on the relevant essential steps to bring about change.

*Figure 1: Theory of Change Map for mhSUN* See Appendix I for Key to Interventions and Assumptions in Theory of Change Map

mhSUN uses a systems approach to address all relevant components of the primary health care system so that potential barriers are considered, and processes are facilitated that lead to better care.

This manual describes the elements that aim to provide sustained services despite the challenges that are often found in similar contexts. It is hoped that the manual will allow those seeking to establish such services in their own districts to do so by learning from the practical experience of this project, and ensure that good evidence-based practice informs their work. This will increase the chances of both providing quality services that have a meaningful impact in peoples’ lives, and increasing the sustainability of such services.

The local context, including the structure of health services and resources available, will rightly inform the character of a local service, but it is important to consider the elements described below, and to have clear reasons for adapting them if necessary. In this way, ‘active ingredients’ that enhance the programme would not be lost.

**Purpose**

The purpose of the intervention is to improve coverage of mental health services at local government level by integrating mental health into PHC services.

This pilot, and the research evaluation associated with it, is expected to provide evidence to support wider roll-out of such integration in Nigeria and other similar contexts.

**Brief service description**

The Federal Neuropsychiatric Hospital in each implementing State (FNPH) will support primary and secondary services to integrate a basic package of mental health care based on the mhGAP Intervention Guide (Nigeria adaptation). The FNPH will provide regular training of personnel, monthly supervision, support for complex cases through referral (stepped care) and specialist outreach clinics (collaborative care) to each District Hospital at least every month.

The mhGAP guide includes algorithms for 8 priority conditions, however under Nigerian health policy, PHC staff are recognised legally as able to provide interventions on depression, psychosis and epilepsy. This is therefore where we will focus our research. According to the Mental Health Policy, Community Health Extension Workers (CHEWs) and Community Health Officers (CHOs) can legally provide first-line medication based on standing orders. These are limited to one medication per condition, but these are in line with mhGAP. During regular supervision at the PHC or District Hospital site, a specialist can modify treatment based on their greater expertise. Few psychological interventions are available at this level, but general principles of care (mhGAP) and psychoeducation will be expected. In addition, local providers of social support will be mapped for referral.

In addition to the work of supporting clinical services, attention will be paid to other system issues, namely governance, health information systems, medication availability, and interaction between the different levels of service (referral and supervision).

The FNPH in each state site will work in a collaborative way with local stakeholders through a representative State Steering Committee. In addition to health service representatives from all levels, this includes relevant community members. Community resources are essential for implementing certain parts of the model, such as the identification of local ‘champions’ for awareness-raising and community support of clients, as well as finding support from other sectors for social, livelihood and human rights interventions.

**Components of the mhSUN Model**

1. The policy and legislative framework for service reform
2. The structure and management of the service
3. Equity and access to the service
4. Human Resources and capacity building
5. Supervision and maintaining quality of care
6. Clinical contact and referrals
7. Maintaining contact, and strategies to deal with non-attenders
8. Availability of medication
9. Raising awareness, and increasing uptake of the service
10. Record-keeping and Health Management Information System
11. Social integration and rehabilitation

## 1. The policy and legislative framework for service reform

Nigeria has made some important steps in recent years towards creating an enabling policy environment for emergence of innovative services to meet the needs of its people. This includes;

- Mental health policy, which has been adopted in 2013^[7]^
- Legislation in late stages of development within FMOH
- Establishment of a national Mental Health Action Committee under FMOH
- Federal Government participation in research projects in collaboration with WHO
- Appointment of a national focal point for mental health in FMOH

Some challenges still remain, for example the constitutional separation of responsibility for health care across the three tiers of government complicates work between the three service levels. Mental health remains a priority in Government and there is a lack of political interest at Federal and State levels. This is reflected in lack of a dedicated budget for community-based or non-specialist hospital services.

There is need to engage with government and gain political support for changes, including having early buy-in so that results are acted upon once results are available.

1.1 All relevant local traditional leadership and political leadership structures should be mapped and a strategy developed to engage with them to communicate their ideal role in supporting the programme (part of Awareness Raising Plan). They will be visited as a courtesy when appropriate during field work and awareness-raising.

1.2 Health system leadership need to be involved in the development of the programme, so as to gain early buy-in to the principle of integration of mental health. They will also be asked to support the process during implementation, with a view to increasing their role towards investment and ownership.

1.3 The programme will be evaluated, and results communicated with local and Federal structures with a view to encouraging investment. At Federal level this will be done through the Mental Health Action Committee and Focal Point for Mental Health. At the State level, health leadership will be engaged in programme development, and the Steering Committee.

## 2. The structure and management of the service

The model is based on integrating mental health into existing structures within the health system, and clinical services remain the responsibility of the relevant tier of government;

- Tertiary care/Federal Neurospsychiatric Hospitals (Federal)
- Secondary care/General Hospitals (State)
- Primary care (LGA)

An ideal balance of care includes services that can meet a variety of needs within these structures. PHC services should be the first point of access for most people, supported by local general hospitals for more complex cases^[8]^. It is now accepted that the few specialists that exist can most effectively work by training and supervising general health workers who provide basic care to the vast majority of patients^[9]^, a process termed task sharing.

The model encourages an active partnership between the zonal specialist hospital (Federal Neuropsychiatric Hospital, Specialist Department at State or Federal Teaching Hospital) with local general hospitals and Primary Care services. These should be involved in development of the programme from the outset, and remain in the Steering Committee.

Non-Governmental Organisations (NGOs) can offer complimentary support to these government services, for example providing some services (eg social, educational, livelihood), relevant technical expertise, and financial support where gaps exist (for example, provision of medication in some contexts). Based on the principle of ‘nothing about us without us’, this programme seeks to ensure that there is a service user voice in the management structures.

2.1 Establish a partnership between LGA-level, State-level, and Federal government structures to oversee development of the programme. NGOs with the relevant expertise can also support this process. This structure would ideally include several interested stakeholders such as service user groups and community organisations in order to ensure it meets the needs of end-users.

2.2 Establish a State Steering Committee with representatives from all stakeholder groups to oversee implementation of the programme, and gain buy-in from relevant authorities. Members include representatives from State MOH, Federal Neuropsychiatric Hospital, LGAs, civil society organisations working in mental health, service users/carers. This Committee should meet at least once every 6 months.

2.3 Role and responsibilities of each actor is clearly defined, and supported by Memorandums of Understanding and contracts where relevant. Heads of institutions are aware of resource implications and support programme. For mhSUN this is outlined below:

*Figure 2: Roles and Responsibilities of Different Actors in the system*

## 3. Equity and ensuring access to the service

The service should be accessible to all members of the community regardless of their social status. This is highlighted in the WHO’s focus on Universal Health Coverage, reinforced in the Sustainable Development Goals. It is necessary to actively work to include those who are often marginalised, such as the very poor, those in rural areas, women, older adults and children, people with disabilities, and those who are hidden or incarcerated because of their mental ill health.

3.1 One of the major barriers to care is the inability to afford transportation to attend the clinics. This is one of the reasons that services are available at the level of primary health care. There should be 5 PHCs with mental health care available in each LGA, with an even distribution through the LGA.

3.2 The service should be affordable, with cost of accessing care in line with other services, and mental health medication available at the facility level no more than 10% above the wholesale purchase price.

3.3 All health information gathered should be disaggregated by age, gender, and impairment so that the degree to which population needs are being met can be measured.

3.4 Community awareness about the service will include efforts to reach all members of the community, including through ‘Community Champions’ in each community.

## 4 Human Resource Competencies and Capacity Building

This is a service that relies on the principle of task-sharing. This means that at each level of the service, the specific tasks expected are clearly defined. Specialist nurses and general doctors are the most appropriate group to provide services at the secondary level, and in Nigeria, CHEWs and CHOs are responsible for care at primary level. General health workers (doctors, nurses, etc) are generally poorly trained in mental health, and those in PHC services are often too busy or unwilling to treat mentally ill clients^[10]^. The result is that at present general service providers rarely feel confident enough to offer mental health services. This model provides (in-service) training so as to build competency to deliver defined tasks at each level, then provides refresher training and supervision to monitor and maintain standards.

The training provided is based on mhGAP, a comprehensive package of evidence-based interventions developed by WHO in collaboration with many implementing countries including Nigeria.

Identification of appropriate personnel is essential. Poor choice of candidates is a common cause of training not having the intended impact. It is important to minimise staff turnover so that the benefits of training are maximised^[11]^, and have stable enough personnel in the programme to build on skills through supervision in the longer term.

4.1 Personnel are identified by the facility for training based on the following principles:

- Once trained, personnel should be available and committed to provide mental health care in the facility. Senior managers should agree to this as a condition of sending staff for training.

- There should be at least one member of staff trained in mhGAP at the facility at any one time once trained. This usually means that there should be a minimum of 3 personnel per facility receiving training.

- They should have proven basic qualification as a CHEW, CHO, registered nurse, or doctor (ie legally allowed to prescribe).

- They should have an interest in mental health care.

- They should be more than 5 years from retirement, and not planning to relocate in the near future.

4.2 A 5 day Training of Trainers (ToT) workshop will be held at the start of the programme in each State, conducted by an experienced team from the University of Ibadan, which has been involved in previous mhGAP-based programmes in Nigeria. 10 people will be trained from each State. These people will be established experts in mental health. The training will focus on the content and teaching techniques for the mhGAP curriculum (published mhGAP training materials will be used including manuals and powerpoint and related audiovisual materials).

4.3 Subsequent to this, in each State, the 5-day mhGAP Base course will be provided using the WHO mhGAP programme training materials that have been adapted for Nigeria (formally endorsed by the FMOH). 50 front-line health staff (CHEWS, CHOs, general nurses and general doctors) will be trained by those trained in the ToT, with a focus on epilepsy, psychosis and depression. A strict list of criteria are used in identification of such staff (above). Each trainee received a copy of the mhGAP Intervention Guide after the training.

4.4 Every 6 months, a one day refresher training on further mhGAP modules is carried out for those who have received the Base Course training.

## 5 Supervision and maintaining quality of care

Supervision is an essential component of the programme. This is provided by qualified mental health professionals (psychiatrists, psychiatric residents, or senior specialist nurses) from the Federal Neuropsychiatric Hospital. Supervision is essential to support those who have received brief mhGAP training, in order to ensure that they carry out their new role to a high standard and in line with evidence-based practice.

The supervision visit should be organized for a regular time, so that the clinic is expecting the visit, and occur monthly at each clinic.

As one purpose of the visit is to see clients and teach while providing these services, the time should coincide with when clinics occur.

5.1 Psychiatrists or psychiatry residents from the FNH in each state will visit the general hospitals every month for provision of supervision and support to general doctors and nurses.

Psychiatrists, psychiatric residents, or psychiatric nurses from the FNH will visit PHC units every month to provide supervision and to support trained CHOs/CHEWs.

5.2 Each supervision visit will include:

- Seeing all the mhGAP trained personnel in the facility and discussing the cases they have seen in the previous month (including using case to review their treatment plans).

- Seeing cases at the facility that day to demonstrate best practice and observe their work. This may include complex cases asked to return to the facility to see a specialist.

- Confirming diagnosis and treatment plan for all new patients by reviewing cases with local clinicians and looking at notes

- Collecting Health Information for the purposes of monitoring, evaluation and research (complete Facility Checklist, using observation and information from Monthly Summary Form, Patient Records etc).

- Checking that the medication supply is sufficient, and the resources necessary to run the service are available (room, mhGAP Guidelines).

5.3 Supervision Report forms will be used to ensure that these elements of the visit are carried out and properly recorded. The supervisor will be responsible for completing the Supervision Report and submitting to the Research Coordinator immediately after every visit.

5.4 In addition, supervision of mental health elements of the service should be included in routine supervision by relevant personnel in PHC and secondary care services. For example, the M&E Officer should also be responsibility for mental health M&E, pharmacy supply chains should be responsible for medication availability etc

## 6 Raising awareness, and increasing uptake of the service

Lack of understanding about mental illness is a problem that acts as a barrier to people suffering from symptoms of mental illness from accessing care. There are many misconceptions that lead to inhumane treatment, and neglect of proper treatment for this vulnerable group. It is therefore important that in addition to providing facility-based services, the community is aware of basic information about mental health, and aware of the availability of services.

6.1 An awareness-raising plan is developed by the programme. This will usually include community events, use of local radio, and sharing of locally appropriate awareness-raising materials (posters and leaflets). The hospital or PHC unit may have experts in community awareness-raising, and mental health can be integrated into other health promotion activities.

See Appendix 2 for Awareness-Raising Template

6.2 It is important to visit and gain the support of local traditional leaders, and make use of established community groups such as women’s groups, age grades and youth groups. Churches and mosques are also a useful way of addressing many community members. There should be a launch event when the programme starts in a facility.

6.3 Community Champions are identified to share basic messages about mental health in communities and to be identified as a point of contact in matters relating to mental health. By providing education to clients, family members and community members, they promote use of the service and discourage human rights abuses. They are also a focus for follow-up in the community where patients lose touch with services.

6.4 The Community Champions will receive training and be fiven awareness resources like the flipchart to educate the population, for example holding town hall meetings, and using forums such as women’s groups, youth, churches, mosques etc

## 7. Clinical care and referrals

Clinical contact with clients should be frequent and comprehensive enough to allow adequate monitoring of progress (particularly benefits and side-effects of medication) but not burdensome to the clients or service. A division of labour ensures that complex cases are seen by more senior staff (psychiatrist), while the majority of routine, stable, cases are managed in a less intense way.

The service aims to provide basic first-line treatment for major priority mental health problems, initially focused on depression, epilepsy and psychosis. The treatments available at the general secondary care hospitals and PHC are in line with the mhGAP-recommended interventions and include;

- Assessment and diagnosis based on mhGAP algorithms.

- Engagement based on respectful, confidential, attentive, non-judgemental, and professional communication (described in more detail in ‘General Principles of Care’ in mhGAP and communicated in training)

- Provision of basic medical treatment (types of medications, and treatment regimes aligned to Standing Orders for CHEWs and mhGAP which has been adopted officially in Nigeria as a strategy for mental health care).

- Provision of sound advice and support to patients and families about maintaining good mental health, avoiding relapse, and engaging better with the community. This includes verbal advice, reinforced with information leaflets (also known as psychoeducation or family education).

- Training of personnel includes basic supportive counselling skills and problem solving therapy (in refresher training).

7.1 New patients and those not responding to treatment will be discussed during supervision sessions, and if necessary invited to be seen during their regular supervision visit to the clinic, or referred to the hospital (FNH or general hospital in mhSUN programme).

7.2 Patients should initially be asked to attend every month, but those whose symptoms are stable can reduce their visits to the clinic to a 2-3 monthly basis for review. Those being seen at a specialist or general hospital should be given the option of being seen at PHC once stable, but they must be given a copy of their Front Sheet, and a referral letter with treatment plan and medication clearly documented.

7.3 Those providing services at the primary and secondary level will be made aware of what care is available at the other levels of care.

7.4 In addition to the basic service offered at the secondary and primary level by general health staff and visiting supervisor, referrals can be made to specialist services available at the Federal Neuropsychiatric Hospital for complex cases of additional interventions stipulated by mhGAP. This includes support for child mental disorders, alcohol and drug use disorders, inpatient care, and more specialised psychological therapies such as Cognitive Behavioural Therapy.

For these referrals, the standard referral form used in PHC centres will be used.

7.5 The availability of community care also allows specialist services to refer to the PHC services for follow-up care of discharged patients.

## 8 Maintaining contact, and strategies to deal with non-attenders

Good follow-up of clients improves compliance with treatment and maintenance of care^[12]^. Therefore, maintaining contact with patients and their carers is central to optimising outcomes. Non-attenders need to be proactively contacted and invited to attend the clinic. The network of Community Champions provides a resource for visiting patients in the community and understanding why they did not attend.

8.1 The patient records should always contain a telephone number for patient and carer, as well as detailed address.

8.2 The Supervisor and local trained clinicians will identify patients who have missed an appointment, and attempt to contact them by mobile telephone, asking them for reasons for this, and inviting them to attend the clinic. If they cannot contact the family, or feel a visit would be useful, they should contact the Community Champion to ask them to visit.

8.3 If the family are neglecting their duties to the client, or there are concerns about human rights abuses, the team should involve the Community Champion, and if necessary, local community leaders, in seeking to persuade them to make appropriate use of the service.

8.4 Community Champions should receive an initial training, where they learn the key facts bout mental health and how to use the flipchart. They should then have 6 monthly refresher and motivation meetings.

## 9. Availability of medication

The psychotropic drugs listed in the standard list (minimum) should be available at each point of service delivery. In general medication is not easily available in private pharmacies and quality can be extremely poor, with high levels of counterfeiting.

Given the importance of medication to comprehensive services, it is the policy in the programme for mhGAP-trained CHEWs, CHOs, nurses and doctors to prescribe. This is in keeping with PHC and Mental Health policy in Nigeria^[13]^. Medical interventions are taught that follow mhGAP guidelines and standards of diagnosis and treatment are maintained through supervision of PHC workers.

It is an absolute necessity that service users are guaranteed a reliable supply of good quality medication^[14]^. In some cases, this will be through standard health structures, but these often do not function well enough, are too expensive, or are not local enough, for clients to access them. Another option is a Drug Revolving Fund managed by the visiting supervision team or external NGO. In this way good quality drugs are supplied by the NGO partner or the hospital to the clinics, sold to the clients at a stipulated cost, and the funds are used to purchase more drugs.

Whatever the system used, the service must ensure the following:

9.1 Prescribed drugs are available at the point of clinical contact for the clients to buy (or be given if the health system allows for free medication). Clinics should never be allowed to run out of drugs (stock-outs, defined as cases where any drug is not available for more than a day).

9.2 Medications available are based on an agreed Essential Drug List. This allows for available, affordable drugs, that are recommended by the WHO in the mhGAP Intervention Guide. Attention is paid to the quality of the drugs, and the most reliable sources are used.

9.3 Clear instructions should be given to clients about the medication, including potential side-effects. The regime of drugs to be taken should be written down, and they should have an adequate supply to last until their next visit.

9.4 The treatment plan is review by the Supervisor on their next visit to the clinic for each new patient (review of medical notes, and if possible, relevant clinician.

## 10. Record-keeping and Health Management Information System

Improving the accuracy and volume of information available for mental health elements of health services provides important tools not only for improved individual care and understanding and adapting the service, but also for advocacy. For example, if the number of patients using the service are recorded and communicated to the relevant authorities, it is possible to ensure that sufficient resources are available for their care, and that the appropriate amount of medication is purchased.

10.1 Each patient with a mental health diagnosis must be recorded on entering the health system, and on every subsequent visit in the mental health register. These figures should be collated in the Monthly Summary form.

10.2 These mental health statistics should be included in the routine health information system reported to PHC and State services, as well as being collected during monthly supervision visits for mhSUN/donor reporting use.

10.3 Each mental health patient should have a ‘Front Sheet’ completed, with unique ID that will be used for tracing records and in all research documentation. See below for how patient ID numbers can be generated.

## 11. Social integration and rehabilitation

Although access to services is often cited by service users as an important priority, it is ultimately their capacity to engage in the life of their communities that they value the most. Other stakeholders in the LGA and State also have an important contribution to make to successful addressing needs of patients and carers in addition to health services.

11.1 The patient and carer will be asked about their priorities during assessment, supportive counselling and psychoeducation. If appropriate they may engage in problem solving therapy to address particular concerns.

11.2 Where useful they may be referred to resources external to the health service for support, such as NGO, faith-based, or government services providing educational, social or livelihood support.

11.3 The Community Champion may be consulted by the medical team to enquire as to social reintegration aspects of treatment. They will be expected to know clients local to their area, and report to the medical team, particularly if there are any major problems.

# Chapter 4: Research Standard Operating Procedures

Consistent and thorough coordination of the research is key to ensuring that all data is collected to a sufficient standard for analysis and accurate results.

Overall coordination of the research is by Julian Eaton, PI, supported by the Advisory Group in London and Nigeria. Research implementation in Nigeria is overseen by the mhSUN coordination team in Ibadan and carried out by the Federal Neuropsychiatric Hospital research teams in Calabar and Kaduna.

The research data collection team is independent of the implementation teams of mhSUN.

In each site, a Research Coordinator oversees the field work of the Research Assistants, and in turn is supervised by a Lead Consultant in each site and the Federal Hospital Chief Medical Director.

Ethical permission is sought from the local sites (Calabar and Kaduna) and in University of Ibadan and London School of Hygiene and Tropical Medicine.

Monthly telephone meetings are held to facilitate smooth running of the research.

## 4.1 Facility Case Study

The Facility Case Study is designed to collect facility/clinic level information about how different elements of the intervention are working, for example staffing, medication availability, organisational structures. This will provide key information for the process research.

1. Prior to the start of the research, the Facility Questionnaire was designed, based on the PRIME PHC questionnaire, and adapted for the Nigerian context by a participatory process involving both teams.
2. It will be tested in each site prior to finalization during the pilot phase.
3. The questionnaire is to be administered by Research Assistants, supervised by the Research Coordinator, in each intervention facility (15 in each site) plus 5 control facilities. This may be done on specific occasions during the routine supervision process.
- At baseline (full questionnaire)
- Quarterly (some information) for one year
4. The information will be collected directly on laptops/tablets using MS Excel. The data will then be stored in accordance to the Data Management procedures.

## 4.2 Routine Health Information from clinics

The clinics will all collect routine information that would form essential data for mental health as an integral part of the overall health system. This will be facilitated (initially) by the mhSUN programme, with a view to this becoming collected internally by the health service as the mental health services become embedded.

1. Routine health statistics should be sent to the relevant authorities as with all other routine statistics.

2. This data will also be collected for the research, and will be used for understanding certain process information, and coverage information. It will generally be collected by health centre staff (on provided documents), and collated for research purposes by Research Assistants during visits (summarized on the Facility Case Study form).

3. In time, it is hoped that the minimal mental health information will be added to future reprints of general health information forms. This is part of the advocacy elements of the programme.

## 4.3 Activity Logs

This is a series of documents that allow tracking of activities so that their potential effect on outcomes can be analysed to investigate processes for change and effective running of a service (as well as to track research implementation). The specific logs include:

- Training (ToT, mhGAP training and follow-up refresher training)
- State Steering Committee Meetings
- Advocacy visits/meetings with political and traditional leaders
- Supervision at clinics
- Facility Case Study completion
- Cohort study field visits and recruitment

For each of these, specified basic information (date, basic outputs etc) are noted.

1. Each form should be completed as a specific activity is carried out by the Research Coordinator.
2. These will be reviewed during monthly telephone planning meetings, during field site visits by JE and occasionally by email.

## 4.4 Cohort Study

The cohort study is designed to follow a number of patients and carers through the identification, treatment and follow-up process. This allows collection of some process information (length of time prior to identification, experience of treatment), as well as some outcome data (disability, symptoms).

*Sample*

Three cohorts of service users (with depression, psychosis and epilepsy), and their linked carers, will be followed from baseline to follow-up at 6 months after recruitment into the study. We will seek to recruit 150 persons in each cohort, based on power calculations for similar studies (450 total).

The main cohort questionnaire is adapted from the PRIME programme^[15]^, with which this research shares several objectives.

Data will be collected by Research Assistants electronically using the Mobenzi Android mobile application. This allows data to be efficiently collected and stored, as well as reducing errors in collection as the design incorporated relevant skips etc.

The questionnaire and methods of data collection in this setting will have been tested during the pilot phase and subsequently adapted as appropriate.

*Sample inclusion and exclusion criteria:*

The sample will consist of people with depression, psychosis, and epilepsy, who are normally resident in identified Local Government Areas in Kaduna and Cross River States.

With a view to quality assurance and efficiency of future replications, the hospital and PHC sites were purposively chosen to meet certain criteria;

- High patient flow rate
- Adequate staff levels to allow one ‘MH personnel’ always on duty
- Balanced geographical coverage of the LGA
- Support from administrative and political leadership structures

We expect to use 15 PHCs per State (average 5 PHCs per LGA). This is a number that the Federal Neuro-Psychiatric Hospital in each State can reasonably provide training, clinical outreaches and supervisory support for, and represents a structure recommended for any subsequent replication.

Note: while this is the sample for the cohort study, all people presenting with a mental health problem to services will be served.

Include*:*

- Men and women diagnosed in primary and secondary services with depression, psychosis or epilepsy
- Ability to speak the language of interviewer
- New patients to the service, or those who have not been in active treatment for at least 6 months.
- Aged between 16 and 65 years for depression and psychosis, and between 5 and 65 years for epilepsy. This is because of the typical ages of presentation of these conditions, increased risk of comorbidity, or complex diagnosis outside of these age ranges.

Exclude:

- Those living outside the LGA running the service. This will reduce attrition. In addition, the assessment of coverage means that patients within the catchment area are of more interest to the study.

Carers are recruited based on these criteria being met for the index patient. A carer is defined as the person attending care with the patient.

*Recruitment:*

PHC staff will contact the research team when patients present (consecutive sampling). In most cases, an independent Research Assistant (RA) will be at the site to assess inclusion criteria, obtain consent, and conduct a baseline interview with the patient and carer. On the next supervision visit, the psychiatrist or psychiatric nurse will confirm diagnosis. If not on site, the team will attempt to find the person in their home and conduct the interview.

We will maximize the number of people who can be recruited directly by attending the clinics during the busiest days at the PHC clinic (often coinciding with market days).

*Consent to treatment*

Consent must be sought from all potential participants prior to interviews. Patient Information Sheets include a summary of the programme and expected use of the data (see Appendix III). However, this information should also be explained to the patient as they are likely to have questions and may not read well. Consent forms are available for patients and carers to sign if they agree after having all the information given to them (Appendix IV).

If there is a concern about the capacity of the client to give consent, the interview can go ahead if the person implies they are willing to participate, but proper consent must be sought at a later date when the person regains capacity. As with any other patient, the person is then free to withdraw their consent at that time (including for information already collected to be used). A similar process will be used with children in the epilepsy study.

**One copy of the signed consent form must be kept for study records and one copy given for patients/carers to take.** A thumb-print will be used as an option for non-literate persons.

A participant may or may not give reasons for deciding not to participate, and it will be clearly explained that this will have no bearing on their receipt of treatment.

*Assigning Unique Identification Numbers (UID)*

The UID is essential to track a patient through the research process (and later for anonymizing data). Every new client will be assigned a number based on the following formula;

- First four numbers based on particular clinic (code sheet is used for this)
- Next four numbers based on patient ID assigned in hospital/clinic registration documentation (usually a serial number)
- Leave out the /16 or similar at the end of the registration serial number

**This must be written on all documentation relating to patients, especially Front Sheet, and during questionnaire completion.**

*Severe Adverse Events*

Each time a patient/carer are interviewed, a number of questions will be asked to ensure that there is no potential risk to the clients that should be taken into account prior to proceeding. These are entitled ‘Severe Adverse Events’.

**If any of these questions (eg risk of suicide, severe medical or psychiatric problem) are answered positively, the Research Coordinator must be informed immediately, and a referral made for assessment by an expert.**

*Follow-up and reduction of attrition:*

After this initial assessment, patients will be seen 6 months and one year later for follow-up assessment by RAs. Where possible, assessment will take place during routine clinic appointments, but if necessary, RAs will find patients at home. All patients will have a unique identifier, and detailed contact information for both patient and carer on both their clinical records and the research documentation.

Based on feedback from the PHC clinic staff, the ‘mental health clinic day’ (where follow-up will occur) should be on a separate day to ensure that there is space for the person to be seen, and time for them to be seen properly. They also felt that this should reduce stigma as it will be relatively quiet, though this remains to be proven.

It is hoped that certain elements of the mhSUN service will themselves reduce attrition, such as the use of telephones to contact clients.

*Questionnaire administration*

The questionnaire should be administered at the same time to the patient and carer. The questionnaire is on a mobile application (works on the supplied android phone or tablet), and for each question it is made clear if it is for the patient or care to answer.

It should take about 40 minutes in total to ask all the questions. The RAs should be very experienced in use of the questionnaire in advance, so that they can efficiently lead the patient and carer through, asking the questions in a clear and precise way.

## 4.5 Qualitative Study

In order to gain a deeper insight into the service from the perspective of different stakeholders, certain groups involved in the service will be interviewed in greater depth to compliment the quantitative studies. Principles of Community-Based Participatory Research (CBPR)^[16]^ will be used to ensure that the issues discussed are influenced by participants. This will inform discussion guides and prompts for those managing the groups, in addition to ensuring that Theory of Change indicators are addressed in relation to the following questions:

a) How acceptable and feasible has the process of scaling up been for various key stakeholders in the health system (providers)?
   - Key informant interviews will be carried out using prompts related to acceptability and feasibility of different aspects of service reform. Key informants will include;
     - Clinicians trained in mhGAP (2 CHEWs, 2 general nurses and 2 general doctors in each state)
     - Supervisors (2 psychiatrists and 2 psychiatric nurses from FNPH in each state)
     - Health service managers (1 PHC Coordinator, 1 District Hospital Manager, 1 Facility Coordinator).
b) What has been the service user, carer, and community experience of the service, particularly in terms of accessibility and acceptability?
   - Purposive samples of patients, carers, and community members involved in the community awareness work will be identified for focus group interviews. Each group will include 5-8 members, and be repeated in the Kaduna and Calabar sites.

Experienced interviewers from local research institutions will be trained by the PI using prepared guidelines in order to maintain focus on key issues. Interviews will be carried out in the local language (Hausa and Efik in Kaduna and Cross River respectively), and recorded. After transcription and translation of recordings, analysis of themes will be carried out using Grounded Theory^[17]^ using computerised analysis software, such as NVivo (used at LSHTM).

# Chapter 5: Data Management Plan

This plan is based on LSHTM’s Data Management Plan (DMP) Online template, available at ([http://dmponline.dcc.ac.uk](http://dmponline.dcc.ac.uk/)).

*Data Collection*

Data for the cohort studies will be collected and entered directly onto an Android mobile device using the Mobenzi application, by trained RAs (5 in each site). They have experience in conducting similar research, and have been provided with the resources to travel, collect and manage data. They are supervised by a local Research Coordinator. The Mobenzi platform is based in South Africa, and has a team with extensive experience in mental health research support in low income settings through the PRIME project and other research^[18]^.

For the Facility Case Studies, paper forms will be used, which will be transcribed onto an electronic format prior to analysis for process indicator changes. Routinely collected information as part of Health Management information System and monthly Supervision Checklists, will also be entered independently following collection by Supervisors into Excel spreadsheets compatible with STATA prior to analysis. This will take place at the State research team offices.

*Data types and formats*

This research is based on a mixed methodology with qualitative and quantitative aspects. Processes of data collection, and choices for storage formats was based on ability to ensure easy access in the long term, while enabling simple local collection and secure transfer. The result is a combination of simple solutions like paper-based forms in clinics, and more advanced alternatives (Android applications). Even the more technological choices were based on ensuring usability in the field, like ability to train users, and to charge devices.

| Data Type | Study | Description of data acquisition | Format | Software |
| --- | --- | --- | --- | --- |
| Quantitative | Cohort | Input onto Android devices during interview with patients and carers at clinic or home setting | Exports as .csv, .dta or .xls | Mobenzi |
|  | Facility Case Study | Baseline and quarterly visits to facilities by Research Assistants. Observation, interview with facility staff, review of documentation | Text and numerical data (.xls) | MS Excel |
|  | Coverage | HMIS forms in clinics collated by M&E Officer, transcribed onto laptop  (outcomes from Cohort study) | Clinic Attendance (.xls) | MS Excel 2010 |
|  | Cost of service | (CSSRI cost data from Cohort study)  Unit data from existing sources (StepCare project) | Analysis (.dta) | STATA-14 |
|  | Process (combination of qualitative and quantitive) | Completion of paper-based Supervision Reports during monthly supervision visits by Psych Resident Doctors and Psych Nurses | Form (.docx)  Analysis (.dta) | MS Word 2010  STATA-14 |
|  |  | Completion of Front Page of clinical records by CHEWs and nurses in clinics | Form (.docx) | MS Word 2010 |
| Qualitative | Acceptability of service | Transcription of audio recorded interviews with patients, carers, health system staff and community members. Documentation of observations, memos, | Audio (.mp3)  Text (.docx)  Analysis (.nvp) | Sound Organiser-1.5  MS Word 2010  Nvivo-11 |
|  | Process | Transcription of audio recorded interviews with patients, carers, health system staff and community members | Audio (.mp3)  Text (.docx)  Analysis (.nvp) | Sound Organiser-1.5  MS Word 2010  Nvivo-11 |

*Data Volume*

The aim is to collect a total of 450 patient/carer dyad entries on the Mobenzi system, at baseline and 6 month time-points.

Facility Case Studies data will be collected from 30 clinics every quarter for 1 year. Monthly HMIS summaries will be collected from 30 clinics (recorded on the same Excel file as the Facility Case Study). Supervision reports will be filed every month for 30 clinics.

30 interviews with key informants and focus groups will be recorded, and transcripts used for the qualitative analysis.

*Data quality*

Mobile device data collection allows close monitoring of quality of data collection in real time, not allowing acceptable or empty responses, and highlighting missing items. Skips are automatically generated.

In advance of the pilot phase, RAs and Research Coordinators will receive training in the Mobenzi application by the PI. In addition, each RA will trial the questionnaire first with colleagues, and then will be observed for the first three patients by the Research Assistant. As all data is uploaded daily after completion, regular monitoring by a remote supervisor (for example site Research Coordinators or the PI) is easy to manage. The devices are basic smart phones, which can hold a charge for a whole day (if dedicated only to this task as we will require). They also allow tracking by GPS, ensuring that the exact location of every interview is recorded. They will be purchased in Nigeria and the research team offices will have facilities to ensure they are charged every evening.

For other paper-based data (Facility Case Studies and clinic HMIS), the Research Coordinator at each site will monitor collection and entry of data by RAs. This is recorded on Activity Logs. Monthly M&E meetings in each site are included in the plan to ensure that this is regularly reviewed, and monthly meetings of the overall research team from all sites by telephone conference have already commenced (July 2015).

*Documentation and Metadata*

The Theory of Change map and related indicator table outlines the purpose and expected analysis of each type of data collected. In addition, the mhSUN model manual (to be developed as part of the PhD) will outline the purpose and use of the M&E data.

Development of a codebook will be a part of the process of analysis for the qualitative research. This will define the allocated codes, including real examples of application and exceptions.

*Ethics, Legal Compliance and Sharing*

Consent forms and Patient Information Sheets include a summary of the expected use of the data, and provision for participants to sign their agreement to this. The application for ethical approval will include a description of how data will be kept confidential in keeping with this agreement with participants.

An annex related to intellectual property rights will be drawn up to complement the existing contract between partners. This will be in line with the policies of the partners and donors involved. In general data is owned by the institution generating it, but the agreement will stipulate that it is free for use for research and teaching use of other partners. Use outside of the partnership will only be with the express written mission of those owning the data.

However, much of the data collected may be useful for other researchers, and where the intended use of the data is in line with the aims of the research, sharing with will be encouraged.

*Storage and Backup*

The Mobenzi database is secure and only accessible by the research team members who possess a password (PI and Research Coordinators at sites). Once data is entered, it is transmitted (by wifi signal or mobile internet) directly to the database held by the Mobenzi company in South Africa. It is not possible (even by Research Assistants) to access the data once entered onto the mobile device. This data is the property of the client (mhSUN) and will be transmitted to LSHTM for analysis when requested. All data for the cohort is anonymised at the point of collection.

Other data collected at the sites will be stored by the Research Coordinator in a password-protected computer, and backed up weekly to a local password-protected hard drive held in the locked project office. It will be sent to a server established for this purpose at LSHTM by the PI at the end of the pilot phase, after baseline data collection, and at the end point of the project.

All the paper forms collected in the project will be held at the two participating hospitals for the duration of the project. Those that form part of the HMIS system will have copies held by the State health system.

In keeping with the LSHTM Records Management Policy, data will be stored in formats acceptable to the UK data Archive (<http://data-archive.ac.uk/create-manage/format/formats-table>), and retained for at least ten years.

*Responsibilities and Resources*

The PI will be primarily responsible for ensuring data management plans are followed, with the collaboration of the participating institutions; Ibadan University, Kaduna and Calabar Federal Neuropsychiatric Hospitals.

The hardware, software, and professional services necessary for data management are included in the mhSUN budget. Training in M&E and supervision of Research Coordinators and RAs will include aspects of their responsibilities related to data management.

| Activity | Role | Name | Responsibilities |
| --- | --- | --- | --- |
| Data capture | Principal Investigator | Julian Eaton | Some elements of data collection; Training RAs and RCs in data capture |
|  | Research Coordinators | Yusuf Akande (Kaduna)  Uche Onukogu (Calabar) | Collecting cohort data from sample  Collecting routine HMIS data from clinics  Collating data collected by clinicians, including Supervisor |
|  | Research Assistants | 5 RAs in each site |  |
|  | Facility M&E Officers | Around 100 facility M&E Officers, clinicians and supervisors | Documenting and summarising patient service use data  Collaborating with Research Assistants and project staff for collection and collation |
|  | Clinicians |  |  |
| Metadata production | Principal Investigator | Julian Eaton | Developing clear manual based on ToC Indicators; Codebook for qualitative data |
| Data quality | Principal Investigator | Julian Eaton | Communicate and visit sites to support RCs. Chair monthly telephone meetings |
|  | Research Coordinators | Yusuf Akande (Kaduna)  Uche Onukogu (Calabar) | Monitor data quality, including by accompanying staff of field trips, training, monthly review meetings.  Enter field-based data onto spreadsheets |
| Storage, Backup and Sharing | Principal Investigator | Julian Eaton | Storing and backing up all data including master dataset onto computer and personal network area at LSHTM |
|  | Supervisor/ Associate Supervisors | Alex Cohen, Oye Gureje, Sujit Rathod | Advising PI on principles and practicalities of data management issues |
|  | Research Coordinators | As above | Inputting data into appropriate formats, regular back-up and transmission |
|  | Chief Medical Directors of Ibadan, Kaduna and Calabar Hospitals | Prof Gureje (I)  Dr Okegbe (C)  Dr Sheikh (K) | Managing data storage processes in their respective hospitals Considering and deciding on data sharing requests, ensuring they are in line with contract and policy of respective institutions |

# Appendix I: Key to Theory of Change Map

1

**Interventions:**

1. Engagement and advocacy with Federal MoH including reporting to MHAC, letters of support etc.
2. Advocacy, training, supervision, referral, evaluation and administration of mhSUN co-ordinated by Federal hospital.
3. State Steering Committee identify suitable general hospitals and PHCs for inclusion in mhSUN, and engage with community structures to get support for mhSUN.
4. General hospitals and PHCs identify motivated staff to participate in training.
5. State Steering Committee include mental health indicators in state HMIS.
6. States to determine best way to ensure drug supply in PHC.
7. Training of Trainers (psychiatrist from Federal Hospital)
8. Training of Medical Officers, CHO, nurses, CHEWs by psychiatrist in mhGAP guidelines.
9. Mapping of community resources by CHEWs and identification of community champions.
10. Awareness-raising, potential case identification & referral to PHC by PHC staff and by community champions.
11. Financial support from community fund to cover costs for the poorest of the poor.
12. Community champions and PHC staff engage with community structures to motivate non-attenders to use services.
13. Psychiatrist from Federal Hospital provides supportive supervision and runs outreach clinics in General Hospital
14. Psychiatric nurses from Federal Hospital provides supportive supervision to PHCs.
15. PHC staff deliver treatment within their capacity and refer those resistant to treatment or complex cases to District Hospital or specialist outreach clinic.
16. District hospital staff deliver treatment within their capacity and refer those resistant to treatment or complex cases to specialist outreach clinic or 3^o^ care.
17. Federal hospital refers stable patients back to PHC.
18. Patients referred to suitable community services to address social, economic and educational needs.

**A**

**Assumptions**

A. Service users exist who are willing and able to sit on the State Steering Committee.
B. States can establish an effective system of ensuring a reliable supply of quality drugs in PHCs.
C. People exist in the community who are motivated to be Community Champions and who have the time and skills to be effective.
D. The programme will be accessible to most vulnerable group of patients (severely ill, chained in the community).
E. PHC staff will be willing to treat people with mental health problems and not refer them all to the general hospital.

# Appendix II: Awareness-raising plan template

**Awareness-raising plan – mhSUN (Draft for adaptation in each state)**

**Phase 1: Engagement with stakeholders**

|  | **Stakeholder** | **Purpose** | **Activity** | **Timeframe** |
| --- | --- | --- | --- | --- |
| 1.1 | Local and State Government  Chairman, Director PHC, HOD Health etc. | To make aware about the programme and gain political buy-in | Visit, agree MOU, share documents/information | Already started – ongoing |
| 1.2 | Traditional leaders  (Specify) | Give their blessing, share information, instructions | Eg in Kaduna; first to Emir for overall support, then gathers all Hakimis (District Heads) and instruct, who gathers and delegates to Dakachi (Village Head), who gathers and delegates to the Mai Ungwar (Ward Heads). |  |
| 1.3 | Religious leaders  Imams, Pastors, Rev Fathers (RC) | To increase their understanding, improve treatment of people with mental illness going to them, and for them to refer cases they see | Describe in layman’s terms common mental disorders. Inform them of closest facility, and they will add a message to sermon or Friday prayers to pass information |  |
| 1.4 | Traditional healers  (Listed in situation analysis) | To ask them to refer people with certain problems to services. Engage with them for dialogue about their practices | Understand their perspective on mental health (causes, treatment etc). Describe in layman’s terms our understanding of mental disorders. Inform them of closest facility, and ask them to refer |  |

**Phase 2: Liaise** with Community Champions/Community groups

|  | **Stakeholder** | **Purpose** | **Activity** | **Timeframe** |
| --- | --- | --- | --- | --- |
| 2.1 | PHC Clinics | CHEWs/other staff integrate MH into regular awareness activities and community mobilization. | Have sessions with PHC staff involved in outreach (or use mhGAP trained staff). Give them flip chart and awareness materials | Already started – ongoing |
| 2.2 | Village Development Committee | Integrate MH into regular health promotion activities of VDC | Add MH to their activities. Information sharing during their regular monthly meeting. Teach them how to identify and refer patients. Tell them which closest facility they can find services nearby. Give flyers/posters | Sep-Dec |
| 2.3 | Community Champions | Expect them to do active case finding, and chase patients who miss appointments. | May be member of VDC or community (identified by VDC). Kaduna likely a woman who can enter all houses. Initially expect a volunteer who has a personal reason to do the work. LATER, they may receive small stipend to cover their expenses. Give them flip chart | Sep-Dec |

Phase 3: Materials development

|  | **Item** | **Purpose** | **Activity** | **Timeframe** |
| --- | --- | --- | --- | --- |
| 3.1 | Flyers | Provide general information to recognize main disorders, human rights, how to find clinic. | Design, translate and print 100 per clinic (1500 per state)  Share during awareness-raising campaigns | By October 2015  After training/pilot phase |
| 3.2 | Posters  (different from mhGAP posters which have been provided by Ibadan) | Promote mhSUN services. Provide general information and direct people to services | Design, translate and print 20 per clinic  (300 per state)  Use during campaign | By October 2015  After training/pilot phase |
| 3.3 | Flip Chart | For use by Community Champions and clinics during awareness-raising  (1 for clinic, 1 for CC) | Design, translate and print (2 per clinic)  Use during campaign | By end Oct 2015  After training/pilot phase |

Phase 4: Awareness campaign

|  | **Item** | **Purpose** | **Activity** | **Timeframe** |
| --- | --- | --- | --- | --- |
| 4.1 | Share awareness materials with PHCs, VDCs/ Community Champions | Allow people in campaign to share information more effectively. Provide permanent access to information for community between campaign events | Distribute and request people working in field to use materials | **After mhGAP training**  End 2015/ throughout 2016 |
| 4.2 | PHC / CHEWs | Use routine awareness / health promotion /community mobilization mechanisms to raise awareness and increase identification and use of service | Include instructions on expected awareness-raising activities in mhGAP training (‘last day’) | **After mhGAP training**  End 2015/ throughout 2016 |
| 4.3 | Community Champions | To have a focal person in community to do awareness raising, someone who community members can go to for information, and who mhSUN can contact to trace patients | Gather together Community Champions and teach how to use Flip Chart | **After mhGAP training**  End 2015/ throughout 2016 |

# Appendix III: Draft cohort participant information sheet

**Participant and Caregiver Information Sheet**

**Study Title: Development and Evaluation of the Mental Health Scale Up Nigeria Pilot Intervention (mhSUN)**

**Part 1**

**1.	Invitation to participate**

You are being invited to take part in a research study. Before you decide whether to participate, it is important for you to understand why the research is being done and what it will involve. Please take time to read the following information carefully. Feel free to ask questions if anything is not clear, or talk to others about the study if you wish.

- Part 1 tells you the purpose of this study and what will happen to you if you take part.
- Part 2 gives you more detailed information about the conduct of the study.

**2.	What is the purpose of the study?**

Mental illnesses are a common cause of disability in Nigeria. Unfortunately, at the moment there are not many services available, and it is expensive and inconvenient to reach services. This study will set up mental health services in normal hospitals and health clinics to see whether they can provide good quality care. If we find that they can provide good care, it may be possible to establish similar types of accessible services in other parts of the country.

**3. What is my role and why have I been chosen?**

You have been chosen to participate in this study because you are someone who is making use of, or may benefit from, the mental health services in the State (or is the caregiver of a service user). We want to know whether the new services that we are offering are useful to you.

**4. Do I have to take part?**

No. It is up to you to decide whether or not to take part. If you do, you will be given this information sheet to keep and be asked to sign a form documenting your willingness to participate.

You are still free to withdraw at any time and without giving a reason. A decision to withdraw at any time, or a decision not to take part, will not affect the standard of care provided to you or your relative.

**5. What will happen to me if I take part?**

If you choose to take part, you will be involved in the study for the next 12 months, which is how long the study lasts.

**6. Treatment and services you will receive**

This project is designed to make the best mental health care available at a centre near where you live and to measure whether you (or your relative) have benefited. It follows the Nigerian Mental health Policy, and recommendations of the World Health Organisation, and is supported by local professionals in the State. The care includes medications, talking therapies and family and social support. All of these treatments are well established and not new. Our main interest is in how we can make them locally available.

**7. Interviews**

In order to understand whether you or the person you care for are benefiting from the services, we want to interview you several times over the course of the research project; once just after agreeing to join the study, and once after 6 months. The interview will be done by a researcher and will be done at your home or another convenient location for you. It will last about 40 minutes.

The interview will include questions on:

- Personal information like age, and marital status.
- Your health and mental health
- Difficulties with social life, work or finances due to the illness
- Challenges you experience
- The amount of time and money you have spent getting treatment

**8. What are the other possible disadvantages and risks of taking part?**

Although there are no new treatments in the new mental health service, and all treatments are recommended by authorities, there is always a risk of side-effects from medication. You will be monitored by professionals, so any negative side-effects of medication should be kept to a minimum.

**9. What are the possible benefits of taking part?**

We cannot promise the new services will improve your illness, or that of your relative, but the services should be more accessible and affordable that previously.

**10.	What happens when the research study stops?**

After the study ends we hope that the service will continue in a similar way, and care will continue to be available locally. If this is not possible, the specialist service available at the Federal Neuropsychiatric Hospital (a partner in this project) will continue to be available.

**11. What if there is a problem?**

You are free at any time to complain or express concern about the way that you or the person you care for has been treated. This will be dealt with promptly by the team conducting the study. More detailed information on this is given in Part 2.

**12. Will my taking part in the study be kept confidential?**

Yes. All the information about your participation in this study will be kept confidential. The details are included in Part 2.

**13. Contact Details**

*[Add contact details – Research Coordinator of State team at the Federal Neuropsychiatric Hospital]*

**This completes Part 1 of the Information Sheet.**

**If having read Part 1, you are considering participation, please continue to read the additional information in Part 2 before making any decision.**

**Part 2**

**14. What will happen if I don’t want to carry on with the study?**

You or the person you care for can withdraw from use of the pilot services at any time. If you do so you can choose whether or not to continue attending the interviews. If you wish, the information collected in your interviews can be destroyed immediately. If you withdraw from using the pilot services and/ or the interviews you will still be able to receive usual care.

**15. What if something goes wrong?**

If you have a concern about any aspect of this study, you should ask to speak to the researchers who will do their best to answer your questions (see contact details at the end of the document). If you remain unhappy and wish to complain formally, you can do this through the project researcher (contact details at the end of the document), or through the Federal Neuropsychiatric Hospital supporting the study in your State.

**16. Will my taking part in this study be kept confidential?**

If you join the study, all information which is collected about you during the course of the research will be kept strictly confidential. Some parts of your medical records and the data collected for the study will be looked at by authorised persons from the University of Ibadan or London School of Hygiene & Tropical Medicine. They may also be looked at by representatives of regulatory authorities to check that the study is being carried out correctly. All will have a duty of confidentiality to you as a research participant and nothing that could reveal your identity will be disclosed outside the research site.

The nurses and doctors in the participating hospitals and clinics will be aware that you are participating in this study.

**17. What will happen to any information I give?**

Information that identifies you will be stored separately and removed from all questionnaires before we analyse the data or report the findings. We will store all information about you securely, on both a computer and in a locked filing cabinet. All private information we collect from you will be kept confidential.

**18. What will happen to the results of the research study?**

The researchers intend to write a report of the results and publish them in a scientific journal. The researchers will hold a meeting in your local area to present the results of the study, which you will be invited to attend. You will not be identified in any report, publication or presentation.

**19. Who is organising and funding the research?**

University of Ibadan, Federal Neuropsychiatric Hospital Calabar, Federal Neuropsychiatric Hospital Kaduna, CBM, and The London School of Hygiene and Tropical Medicine, are organising this study. The Australian Government and CBM are funding the study.

**20.	Who has reviewed the study to make sure it is safe and appropriate?**

This study was given a favourable ethical opinion by the Ethical Review Board of the Federal Neuropsychiatric Hospital and by experts at London School of Hygiene and Tropical Medicine.

**You will be given a copy of the information sheet and a signed consent form to keep.**

**Thank you for taking the time to read this sheet**

# Appendix IV: Draft cohort consent form

**Title of Project: Development and Evaluation of the Mental Health Scale Up Nigeria Intervention (mhSUN)**

**Name of Principal Investigator: Dr Julian Eaton**

**Name of local Research Coordinator: ________________**

| **Please initial box** | |
| --- | --- |
| 1. I confirm that I have read and understand the subject information sheet dated ......….) for the above study. I have had the opportunity to consider the information, ask questions and have had these answered fully. | ⬜ |
| 2. I understand that my participation is voluntary and I am free to withdraw at any time, without giving any reason, without my medical care or legal rights being affected. | ⬜ |
| 3. I understand that sections of any of my medical notes and data collected during the study may be looked at by responsible individuals from University of Ibadan, London School of Hygiene and Tropical Medicine or from regulatory authorities where it is relevant to this research. I give permission for these individuals to access my records. | ⬜ |
| 4. I agree to the health centre being informed of my participation in the study. | ⬜ |
| 5. I agree to take part in the above study. | ⬜ |

________________________	________________

Name of Participant	Signature [or thumbprint]	Date

_________________________	________________

Name of Person taking consent	Signature	Date

1 copy for participant; 1 copy for research project records; 1 (original) copy to be kept in health centre notes

**References**

1. Lancet Global Mental Health Group. Scale up services for mental disorders: a call for action. Lancet 2007; 370: 1241–52
2. WHO World Health Report, 2001: New Understanding, New Hope. WHO: Geneva, 2001
3. World Health Organization. Mental Health Gap Action Program (mhGAP): scaling up care for mental, neurological and substance abuse disorders. Geneva: World Health Organization, 2008
4. Esan O, Abdulmalik J, Eaton J, Kola L, Fadahunsi W, Gureje O. Mental Health Care in Anglophone West Africa. Psychiatric Services 2014; 65(9):1084-1087
5. Abdulmalik J, Fadahunsi W, Kola L, Nwefoh E, Minas H, Eaton J and Gureje O. The Mental Health Leadership and Advocacy Program (mhLAP): a pioneering response to the neglect of mental health in Anglophone West Africa. Int Journal of Mental Health Systems 2014; 8:5. doi:10.1186/1752-4458-8-5
6. Eaton J, McCay L, Semrau M, Chatterjee S, Baingana F, Araya R, Ntulo C, Thornicroft G, Saxena S. Scale up of services for mental health in low-income and middle-income countries. Lancet. 2011; 378(9802):1592-603
7. Federal Ministry of Health Nigeria. National Policy for Mental Health Service Delivery: FOMH, Abuja 2013
8. Thornicroft G and Tansella M. Balancing Community-based, and hospital-based mental health care. World Psychiatry 2002; 1(2):84-90
9. Kakuma R, Minas H, van Ginneken N, Dal Poz M, Desiraju K, Morris J, Saxena S, Scheffler R. Human resources for mental health care: current situation and strategies for action. Lancet 2011; 378: 1654–63
10. Muga FA, Jenkins R. Public perceptions, explanatory models and service utilisation regarding mental illness and mental health care in Kenya. Social Psychiatry and Psychiatric Epidemiology 2008, 43:469–476
11. Cohen A, Kleinman A, Saraceno B. World mental health case book: social and mental health programs in low-income countries. New York, NY, USA: Kluwer, 2002
12. Chatterjee S, Patel V, Chatterjee A, Weiss HA. Evaluation of a community-based rehabilitation model for chronic schizophrenia in rural India. British journal of Psychiatry 2003;182:57-62
13. Available at www.mindbank.org
14. Eaton J. Ensuring access to psychotropic medication in sub-Saharan Africa. African Journal of Psychiatry 2008; 11(3): 179-181
15. Lund C, Tomlinson M, De Silva M, Fekadu A, Shidhaye R, Jordans M, et al. PRIME: A Programme to Reduce the Treatment Gap for Mental Disorders in Five Low- and Middle-Income Countries. PLoS Med 2012; 9(12): e1001359. doi:10.1371/journal.pmed.1001359
16. Horowitz CR, Robinson M, Seifer S. Community-based participatory research from the margin to the mainstream: are researchers prepared? Circulation 2009; 119(19):2633-42
17. Strauss A, Corbin J. Basics of Qualitative Research: Grounded Theory Procedures and Techniques. Sage; 1990.
18. [www.mobenzi.com](http://www.mobenzi.com/)
