# Supplementary material for: Protocol for process evaluation of integration of mental health into primary healthcare in two states in Nigeria: the mhSUN programme
Source: BJPsych Open. 2021 Feb 15;7(2):e54. doi: 10.1192/bjo.2021.7 (PMC8058900; doi:10.1192/bjo.2021.7)
Supplement: Supplementary file 1 [file bjosup.zip › S2056472421000077sup001.zip!/Appendix_3._Process_Flow_Diagram.docx]

**Appendix 3: Process Flow Diagram**

Patient reaches services - Awareness-raising programme - Availability of service at facility

Adequately assessed - Training and supervision - Guidelines/technical resources

Appropriate treatment prescribed - Supervision review - Guidelines/technical resources

Treatment received as prescribed - Adequate resources at facility (including medications) - Psychoeducation (and leaflets)

Follow-up care - Follow-up appointments - Identification of non-attenders and making contact

Contact coverage

- Proportion of the population in need who access services

Referral

- Mental Health

- Other health

- Other sector (inc community)

Effective coverage

- Proportion of population in need who reach recovery criteria (at 6 months)

Adequate coverage

- Proportion of population in need who receive correct care
